# Real-Time Bio-Inspired Polarization Heading Resolution System Based on ZYNQ Heterogeneous Computing

**DOI:** 10.3390/s25092744

**Published:** 2025-04-26

**Authors:** Yuan Li, Zhuo Liu, Xiaohui Dong, Fangchen Dong

**Affiliations:** School of Information and Communication Engineering, North University of China, Taiyuan 030051, China; 18834878986@163.com (Z.L.); s202405057@st.nuc.edu.cn (X.D.); dongf343@gmail.com (F.D.)

**Keywords:** polarized light, polarized navigation sensor, ZYNQ, heading angle solution

## Abstract

Polarization navigation is an emerging navigation technology, that exhibits significant advantages, including strong anti-interference capability and non-cumulative errors over time, making it highly promising for applications in aerospace, autonomous driving, and robotics. To address the requirements of high integration and low power consumption for tri-directional polarization navigation sensors, this study proposes a system-on-chip (SoC) design solution. The system employs the ZYNQ MPSoC (Xilinx Inc., San Jose, CA, USA) as its core, leveraging hardware acceleration on the Programmable Logic (PL) side for three-angle polarization image data acquisition, image preprocessing, and edge detection. Simultaneously, the Processing System (PS) side orchestrates task coordination, performs polarization angle resolution, and extracts the solar meridian via Hough transform. Experimental results demonstrate that the system achieves an average heading angle output time interval of 9.43 milliseconds (ms) with a mean error of 0.50°, fulfilling the real-time processing demands of mobile devices.

## 1. Introduction

In the era of rapidly advancing navigation technologies, there is a growing demand for high-precision and highly integrated navigation sensors in aerospace, autonomous driving, and robotics. Traditional navigation systems such as GPS and inertial navigation systems (INS) exhibit inherent limitations. For example, GPS signals are prone to signal blockage or electromagnetic interference, whereas INS errors progressively accumulate over time. Polarization navigation, an emerging technology, has garnered significant attention in the navigation field owing to its advantages, including robust anti-interference capability and non-cumulative errors over time [1].

Since the 1960s, the team led by Wehner at the University of Zurich has pioneered research into the polarized light navigation mechanisms of desert ants and honeybees, establishing the foundational feasibility of polarization-based navigation [2]. Building on this work, Lambrinos et al. [3,4] developed polarization detectors inspired by the polarization-sensitive neural structures of ant compound eyes. They proposed a novel visual landmark navigation model, further validating the practicality of polarization navigation. Concurrently, Labhart [5] advanced this field by constructing an optoelectronic model neuron to simulate the physiological properties of cricket polarization-opponent interneurons (POL-neurons). His work rigorously evaluated the intensity and directional stability of sky polarization signals under varying meteorological conditions, confirming their reliability as navigational references. In parallel, Chu Jinkui’s team designed a six-channel bio-inspired polarization navigation sensor based on photodiodes [6], which was subsequently deployed and tested on mobile robots [7]. Yong et al. [8] refined mechanical rotary polarization imaging systems, significantly improving the polarization imaging speed and laying the groundwork for dynamic target detection. Between 2015 and 2016, a research group led by Prof. Hu Xiaoping at the National University of Defense Technology [9] developed three generations of polarization imaging devices. The first-generation system employed a single optical camera with a rotatable graduated polarizer to capture polarization-direction-specific images. The second iteration utilized four optical cameras, each equipped with linear polarizers at 0°, 45°, 90°, and 135°, enabling quad-channel polarization image acquisition using USB 3.0 [10]. By 2018, the team introduced a third-generation microarray polarization imaging device that integrated a quadrant polarizer above a CCD sensor to simultaneously capture multidirectional polarization images with a single camera [11].

Recent advancements include Liu et al.’s [12] 2022 miniaturized bio-inspired polarization sensor, which mimics the compound eyes of insects. Their design incorporated a multithreshold segmentation algorithm to filter invalid pixels and a least-squares method to compensate for intrinsic errors, achieving calibration accuracies of ±0.3° indoors and ±0.5° outdoors. In 2023, Wenhong et al. [13] designed a tri-channel bio-inspired polarization sensor based on desert ant polarization perception mechanisms. This sensor employs orthogonally aligned polarizer-covered photodiodes combined with logarithmic amplifiers to achieve compact structure, real-time performance, and low cost. The experimental results demonstrated a maximum polarization angle error of ±3.9° and a mean error of 1.53°, highlighting its practical viability. Recent advancements in multiscattering models, such as the Equivalent Incident Light (EIL) framework coupled with simulated annealing optimization, have improved environmental resilience, achieving sub-0.4° heading accuracy across diverse scenarios [14]. Nevertheless, hardware imperfections, including polarizer misalignment and detector response non-uniformity, introduce additional errors, necessitating precise calibration strategies [15].

Existing rotary polarization cameras are prone to mechanical failures during prolonged operation and suffer from sequential polarization data acquisition across multiple orientations, which severely compromise the real-time responsiveness of the system. Furthermore, multichannel polarization sensors exhibit excessive reliance on host computers for image processing, impeding system integration and miniaturization while limiting their applicability in portable mobile devices. To address these challenges and align them with the demands of systematic design and mobile deployment, this study proposes a system-on-chip (SoC) solution tailored for tri-directional polarization navigation sensors, emphasizing high integration and compact form factors. The system employs a ZYNQ MPSoC as the core controller to enable the real-time acquisition and resolution of sky polarization information. To enhance real-time performance, hardware acceleration via field programmable gate array (FPGA) was implemented for image acquisition, preprocessing, and Canny edge detection. This architecture achieves sensor miniaturization and integration without sacrificing navigation accuracy, thereby paving the way for its deployment in portable mobile platforms.

## 2. Materials and Methods

### 2.1. Polarization Navigation Principle

Atmospheric-scattered light is predominantly partially polarized. In the field of atmospheric polarization detection, the polarization state of any light can be described using the Stokes vector, expressed as

(1) $$S={\left[I,Q,U,V\right]}^{T}$$

where *I* is the total light intensity, *Q* is the horizontal linear polarization component, *U* is the linear polarization component at 45°, *V* is the circular polarization component.

In natural environments, the circular polarization component (*V*) is typically negligible because of its minimal intensity.

The intensity of the outgoing polarized light $I^{\prime }(\theta )$ can be related to the Stokes vector of the incident light using the following expression:

(2) $$I^{\prime }(\theta )=\frac{1}{2}\left(I+Q\cos2\theta +U\sin2\theta \right)$$

By measuring the light intensities at three distinct polarization angles (0°, 45°, and 90°), the Stokes vector components can be derived as

(3) $$\left\{\begin{array}{c} I=I^{\prime }\left(0{}^{\circ}\right)+I^{\prime }(90{}^{\circ})\\ Q=I^{\prime }\left(0{}^{\circ}\right)-I^{\prime }(90{}^{\circ})\\ U=2\times I^{\prime }\left(45{}^{\circ}\right)-I^{\prime }\left(0{}^{\circ}\right)-I^{\prime }(90{}^{\circ}) \end{array}\right.$$

The degree of polarization (*Dop*) and polarization angle (*Aop*) of the incident light were calculated as [16]

(4) $$Dop=\frac{\sqrt{Q^{2}+U^{2}}}{I}\;Aop=\frac{1}{2}\tan^{-1}\left(\frac{U}{Q}\right)$$

The polarization azimuth angle (*AOE*), defined as the angle between the polarization direction vector and the local meridian tangent in the sky observation coordinate system, was computed as follows:

(5) AOE=Aopi,j−tan−1⁡i−h2j−w2    −90°≤AOEi,j≤+90°Aopi,j−tan−1⁡i−h2j−w2−180°        AOEi.j>+90°Aopi,j−tan−1⁡i−h2j−w2+180°        AOEi,j<−90°

In Equation (5), *(i*, *j*) denotes the pixel coordinates, *h* represents the image height, and *w* corresponds to the image width [17].

**Figure 1:**
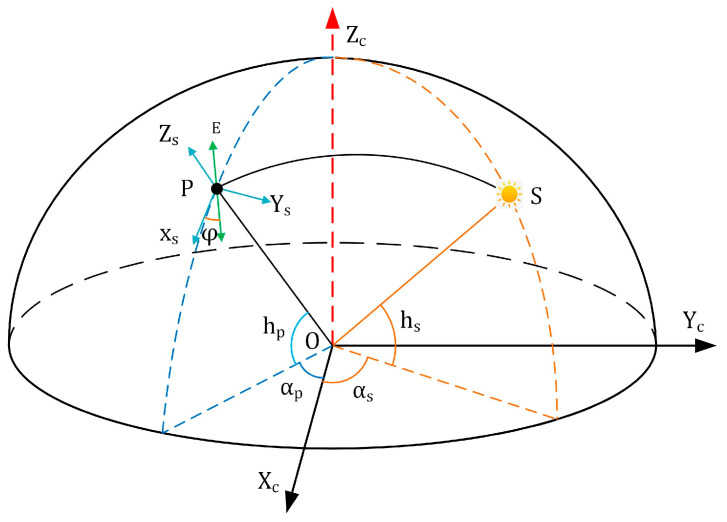
Schematic diagram of the celestial coordinate system. O represents the observer’s position, S denotes the solar position on the celestial sphere, P indicates the observation point. O−X_c_Y_c_Z_c_: polarized camera coordinate system. P−X_s_Y_s_Z_s_: scattered light local coordinate system.

The fundamental principle of polarized light navigation involves obtaining the polarization azimuth angle at the zenith point, determining the angular relationship between the observer and the solar vector, and ultimately resolving current heading information. As illustrated in Figure 1, a coordinate system is established with the sensor as the origin O, projecting both the Sun (S) and the sensor’s observation point (P) onto an imaginary celestial sphere. Here, h_s_ denotes the solar elevation angle, a_s_ represents the solar azimuth angle, h_p_ indicates the observation point’s elevation angle, and a_p_ signifies the observation point’s azimuth angle. Since the E-vector (polarization direction) is perpendicular to the scattering plane (OPS), their cross-product relationship is expressed as

(6) $$E_{c}=OS_{c}\times OP_{c}$$

In this formulation, *E_c_* represents the E-vector at the observation point within the polarization camera coordinate system. The scattered light vector can be expressed as $OP_{c}={\left[\begin{array}{ccc} \cos h_{p}\cos a_{p} & \cos h_{p}\sin a_{p} & \sin h_{p} \end{array}\right]}^{T}$ and the solar illumination vector is defined as $OS_{c}={\left[\begin{array}{ccc} \cos h_{s}\cos a_{s} & \cos h_{s}\sin a_{s} & \sin h_{s} \end{array}\right]}^{T}$.

The transformation matrix $C_{c}^{s}$, which maps coordinates from the polarization camera system to the local scattering light coordinate system, is given by

(7) $$C_{c}^{s}=\left[\begin{array}{ccc} \sin h_{p} & 0 & -\cos h_{p}\\ 0 & 1 & 0\\ \cos h_{p} & 0 & \sin h_{p} \end{array}\right]\left[\begin{array}{ccc} \cos a_{p} & \sin a_{p} & 0\\ -\sin a_{p} & \cos a_{p} & 0\\ 0 & 0 & 1 \end{array}\right]$$

The E-vector *E_s_* at the observation point in the local scattering light coordinate system can then be expressed as

(8) $$E_{s}=C_{c}^{s}\times E_{c}$$

Substituting Equations (6) and (7) into Equation (8) yields

(9) $$E_{s}=\left[\begin{array}{c} \cos h_{s}\sin\left(a_{s}-a_{p}\right)\\ \cos h_{p}\sin h_{s}-\sin h_{p}\cos h_{s}\cos\left(a_{s}-a_{p}\right)\\ 0 \end{array}\right]$$

The direction of the E-vector *φ* in the local coordinate system can be represented in two dimensions as

(10) $$\tan\varphi =\frac{\cos h_{p}\sin h_{s}-\sin h_{p}\cos h_{s}\cos\left(a_{s}-a_{p}\right)}{\cos h_{s}\sin\left(a_{s}-a_{p}\right)}$$

where *φ* denotes the azimuth angle of the electric field vector (i.e., the polarization azimuth angle, *AOE*) in the local meridian coordinate system. Here, *h_s_* represents the solar elevation angle, *a_s_* the solar azimuth angle, *h_p_* the observation point’s elevation angle, and *a_p_* the observation point’s azimuth angle [18].

Comparative analysis of the distribution characteristics between the degree of polarization (*Dop*) and the polarization angle (*Aop*) revealed that the *Aop* distribution exhibited relatively low sensitivity to climatic variations. Its distribution pattern consistently aligns with the solar meridian as the symmetry axis, demonstrating robust resistance to external atmospheric disturbances. Consequently, the stability inherent in the *Aop* distribution makes it an ideal information source for polarization-based navigation systems [19].

**Figure 2:**
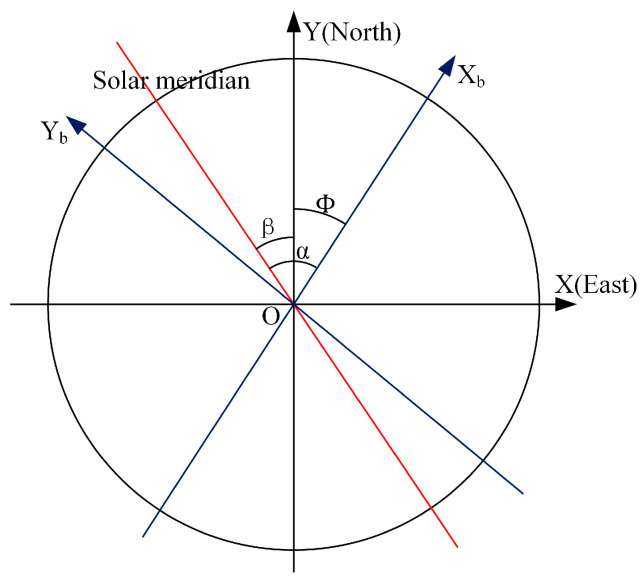
Diagram of the heading angle calculation.

Definition and calculation of heading angle: The heading angle is defined as the horizontal angular deviation between the vehicle’s orientation and true north. As depicted in Figure 2, the geographic coordinate system serves as the reference frame, with the circle center O representing both the observation position and the vehicle’s centroid. The *X*-axis and *Y*-axis align with true east and true north directions, respectively. The body-fixed axis of the vehicle coordinate system is denoted as X_b_. The solar azimuth angle α in the vehicle coordinate system can be derived from the slope k of the solar meridian within this frame. The solar azimuth angle *β* in the geographic coordinate system is calculated using position-time data from navigation/timing equipment combined with astronomical ephemeris. The vehicle heading angle *Φ* is then determined through Equations (11) and (12).

(11) tan⁡α=k

(12) ϕ=β−α

### 2.2. System Architecture

**Figure 3:**
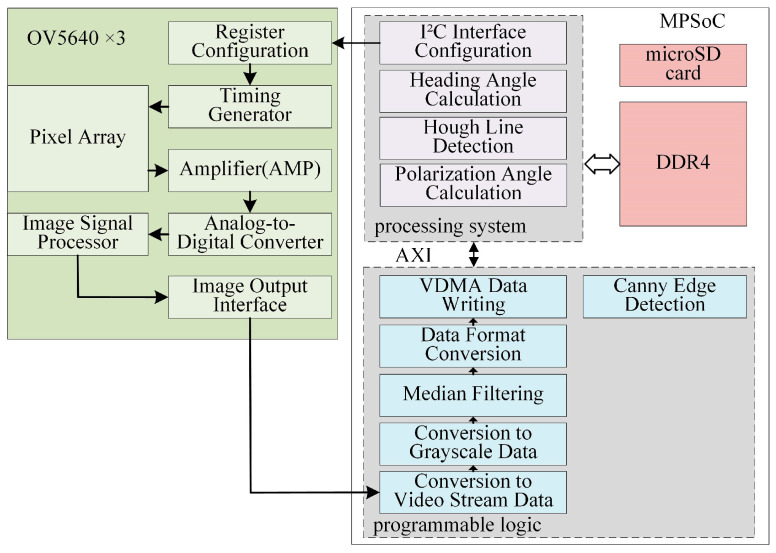
System block diagram of the polarization navigation sensor (vdma: Video Direct Memory Access).

The proposed system comprises seven key components: a tri-channel polarization image acquisition module, image acquisition control module, image preprocessing module, polarization azimuth angle resolution module, Canny edge detection module, and Hough-based heading angle resolution module. The overall system block diagram of the polarized light navigation sensor is shown in Figure 3.

This study employed the Xilinx ZYNQ MPSoC as the core processing unit, which integrates Programmable Logic (PL) and a Processing System (PS, ARM-based). The PL subsystem controls three CMOS cameras (ALIENTEK, Guangzhou, China) to simultaneously acquire polarization image data at three distinct angles. It also executes real-time image-preprocessing tasks, including

1. Data Format Conversion: Transforming 8-bit pixel data (synchronized by horizontal and vertical signals) into video stream formats;
2. Grayscale Conversion: Reducing computational complexity for downstream processing;
3. Median Filtering: Eliminating isolated noise points to enhance pixel value fidelity.

The preprocessed data are transmitted to the PS subsystem via the AXI bus. The PS manages the DDR4 memory for data storage/retrieval and performs polarization angle resolution. Specifically, the Hough transform [20] was applied to extract the solar meridian to determine the heading angle.

### 2.3. Sensor Architecture Design

The sensor employs a trinocular bio-inspired visual architecture that combines a triangular configuration with polarization optics fusion to achieve high-precision environmental perception and navigation. The core structure consists of three independent optical modules positioned at the vertices of an equilateral triangle. Each module integrates a vertically stacked assembly of CMOS camera, polarizer, and narrowband optical filter. The inter-module spacing is optimized to maximize the field-of-view (FOV) overlap while minimizing multi-perspective distortion.

The inter-module spatial configuration refers to the physical spacing between three independent optical modules (CMOS cameras). This design achieves dual objectives: optimizing the field-of-view (FOV) overlap ratio while mitigating multi-perspective distortion effects. Through precise spacing adjustment, the system ensures sufficient FOV overlap across three channels for high-precision data fusion, while preventing geometric distortions or redundant information interference caused by excessive module separation.

Optimization methodology: A high-precision calibration target (checkerboard pattern) is positioned perpendicular to the sensor plane. Tri-channel image acquisition is performed, followed by feature point matching to quantify: FOV overlap error and distortion coefficients

The spacing is optimized using the least squares method to minimize the distortion coefficient.

Field-of-View (FOV) Design Rationale and Measurement Methodology: the theoretical calculation of FOV requires precise correspondence between the effective imaging area dimensions of the sensor and the optical system parameters. According to optical imaging principles, the mathematical expression for three-dimensional field of view angles can be formulated as

(13) $${FOV}_{\theta }=2\tan^{-1}\left(\frac{d_{\theta }}{2f}\right)\;\;\theta \in \left\{h,v,d\right\}$$

where *FOV_h_*, *FOV_v_*, and *FOV_d_* represent the horizontal, vertical, and diagonal field of view angles of the sensor, respectively. The parameters *d_h_*, *d_v_*, and *d_d_* correspond to the horizontal width, vertical height, and diagonal length of the sensor’s effective imaging area, while *f* denotes the effective focal length of the lens.

To validate the accuracy of the theoretical model, we established an experimental system incorporating a standardized checkerboard calibration target. The experimental configuration involved

1. Positioning a grid target (grid spacing L) perpendicular to the optical axis;
2. Maintaining a controlled test distance D between the target and the lens;
3. Capturing grid pattern images through the optical system;
4. Extracting effective imaging boundaries from acquired images.

Imaging scale factors were calculated using the following equation:

(14) $$k_{\theta }=\frac{n_{\theta }\cdot L}{N_{\theta }\cdot p_{\theta }}$$

where *n_θ_* denotes the number of complete grid units in the image, *L* represents the physical grid spacing (mm), *N_θ_* corresponds to the total pixel count in the corresponding direction, and *p_θ_* indicates the physical pixel dimension. Through empirical data calibration of the theoretical model, the experimental field of view angles were determined as

(15) $${FOV}_{\theta ,exp}=2\tan^{-1}\left(\frac{k_{\theta }\cdot d_{\theta }}{2D}\right)$$

**Figure 4:**
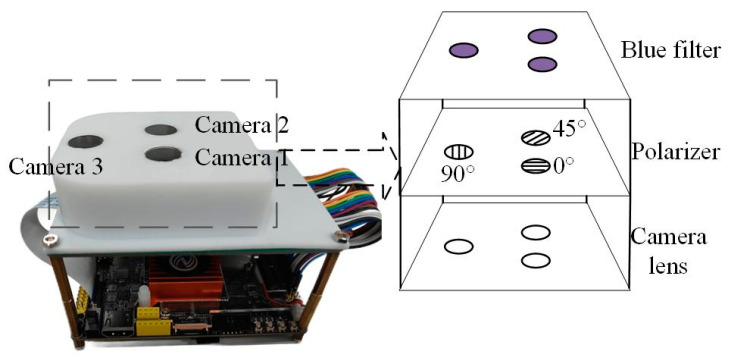
Tri-channel polarization-based navigation sensor.

The CMOS camera utilizes an OV5640 image sensor with a 1280 × 800 resolution and a 70° × 70° FOV. The imaging system is equipped with a 1/4-inch optical lens featuring an F2.8 aperture. The lens configuration delivers a 70° field of view (FOV) with an effective focal length of 3.34 mm. Polarizers, fabricated via nanoimprint lithography, adopt wire-grid structures and are orthogonally aligned at 0°, 45°, and 90° across the three channels. This configuration enables the synchronous capture of three orthogonal polarization components, providing complete polarization state inputs for the Stokes vector computation. Optical filters employ bandpass coating technology with a central wavelength matched to the dominant peak of skylight polarization (450 ± 10 nm), effectively suppressing ambient stray-light interference [21]. A photograph of the tri-channel polarization navigation sensor is shown in Figure 4.

### 2.4. Trinocular Acquisition System Design

The image acquisition module employed an OV5640 image sensor with a photosensitive array resolution of 2592 × 1944 (5-megapixel). Utilizing OmniVision’s (OmniVision Technologies, Inc., Santa Clara, CA, USA) OmniBSI (Backside Illumination) technology, the sensor achieved enhanced quantum efficiency and low-light performance. The configuration of internal sensor parameters, such as exposure time and resolution, is performed via the I^2^C Inter-Integrated Circuit (IC) protocol interface.

**Figure 5:**
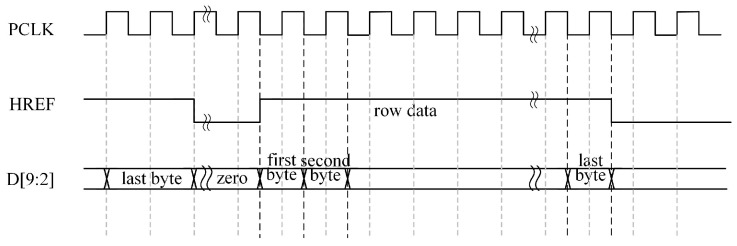
Timing diagram for RGB565 output format.

OV5640 outputs up to 10-bit data, though only the upper eight bits are valid in the RGB565 output mode. The pixel data become valid when the Horizontal Reference (HREF) signal is high. As shown in Figure 5, the first transmitted byte contains the upper eight bits of the RGB565 data, followed by the second byte carrying the lower eight bits. These two bytes combine to form a complete 16-bit RGB565 pixel value [22]. Notably, data transitions occurred at the falling edge of the pixel clock (PCLK). To ensure stable sampling, data acquisition was synchronized with the rising edge of the PCLK, thereby capturing values in their most stable state.

Following successful image acquisition, the raw image data were converted into a video stream format via the image acquisition IP core. Subsequently, the video stream underwent grayscale processing, in which the RGB565 data format was transformed into the YCbCr format. This conversion alleviates the computational load on the post-processing unit (PS side) and enhances the real-time processing capability of the system. The grayscale-converted data were then subjected to median filtering to suppress the transient noise.

**Figure 6:**
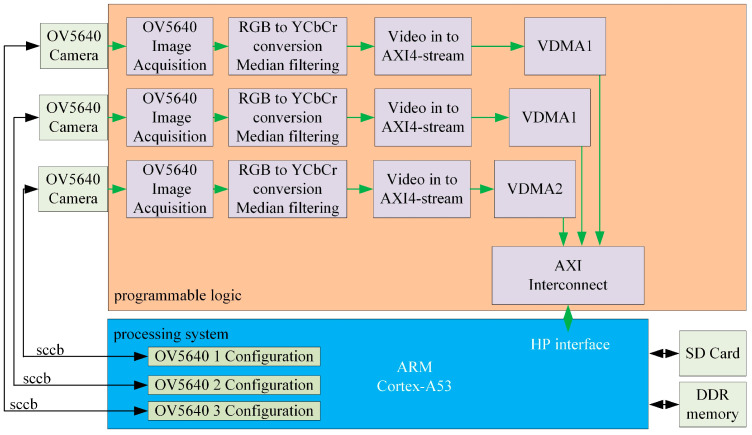
System architecture diagram.

The filtered video stream is routed through a Video-in to AXI4-Stream IP core, converting it into an AXI4-Stream-compliant data flow. This stream is further processed by the write channel of the Video Direct Memory Access (VDMA) controller, which translates the data into an AXI4 Memory Map format for storage in DDR4 memory. VDMA interfaces with DDR4 via the AXI SmartConnect IP core and AXI_HP (High-Performance) ports, enabling efficient memory access and seamless data exchange between the image processing pipeline and storage subsystems. The architectural block diagram of this system is shown in Figure 6.

Synchronized image acquisition for triple-OV5640 sensor array: The synchronized capture of the triple-camera system is governed by a button-triggered interrupt mechanism. During the preprocessing phase, the system executes image acquisition, processing, and data format conversion following the workflow detailed in Figure 6. This sequence culminates in activating the VDMA write channel to transfer image data into the frame buffer.

Upon each button press, the system simultaneously retrieves current-frame images from all three VDMA controllers, storing them at predefined addresses within the DDR4 memory. Subsequent processing stages fetch image data from these designated memory locations for computational operations.

### 2.5. Processor System Design

#### ZYNQ Heterogeneous Computing Mechanism and Advantages

The ZYNQ Multiprocessor System-on-Chip (MPSoC) achieves hardware–software co-optimization through its heterogeneous architecture-integrating Programmable Logic (PL, FPGA) and Processing System (PS, ARM). The core technological benefits manifest in three operational dimensions:

On the PL side, computationally intensive real-time tasks are accelerated through pipelined execution, including

- Synchronized tri-channel polarized image acquisition;
- Preprocessing pipelines (grayscale conversion, median filtering);
- Hardware-accelerated Canny edge detection.

The parallel architecture of FPGAs enables simultaneous processing of multi-channel data, significantly reducing task latency. The PS side orchestrates algorithm execution (Stokes vector computation, Hough transform) and system control logic, leveraging the ARM processor’s advantages in

- Flexible task scheduling;
- Memory bandwidth management;
- Power-efficient computation.

The on-chip heterogeneous architecture of ZYNQ significantly reduces computational load through hardware acceleration while minimizing data transfer overhead between memory hierarchies via efficient PL-PS collaboration. Experimental results demonstrate the ZYNQ system’s total power consumption of 3.6 W, representing merely 1/7 of PC-based implementations (25.95 W). Moreover, the integrated design of PL and PS eliminates the need for external components (e.g., standalone FPGA boards or coprocessors), significantly reducing the system’s physical footprint and aligning with the compactness requirements of mobile devices. 

Comparative Analysis: Without employing a heterogeneous architecture and relying solely on general-purpose processors (e.g., PCs) to execute all tasks, image preprocessing and other operations would require software implementation, resulting in significantly increased processing time. Experimental results demonstrate that the average response time reaches 2743.21 ms on PC platforms, whereas the ZYNQ system requires merely 9.43 ms. Furthermore, the absence of an FPGA would constrain synchronous acquisition of tri-channel data and real-time processing capabilities, rendering the system’s latency inadequate for mobile devices’ real-time requirements. Adopting a discrete architecture (e.g., separate FPGA+ARM configuration) would necessitate additional communication interfaces and power management modules, consequently increasing power consumption and system complexity. Although a pure software solution (ARM-only implementation) simplifies hardware design, it fails to satisfy both real-time performance and energy efficiency requirements.

**Figure 7:**
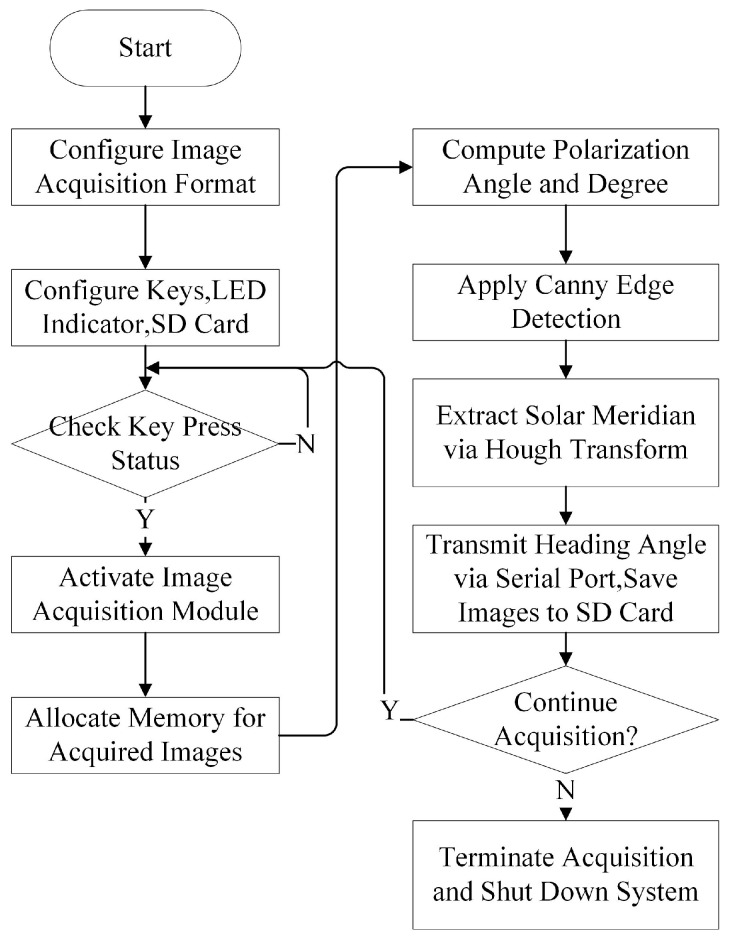
Operational flow chart of sensors.

The primary responsibilities of a Processing System (PS) include controlling the image sensor and managing intellectual property (IP) cores within the FPGA. The system utilizes the Xilinx Vitis (2020.2) software platform, which supports embedded software development and accelerated application deployment across Xilinx heterogeneous computing platforms (e.g., FPGA, System-on-Chip (SoC), and a verse-adaptive compute acceleration platform (ACAP)). Figure 7 shows an operational flowchart of the sensor.

Initialization Phase

1. Configuration Setup: Image acquisition parameters (e.g., resolution and frame rate) are defined using camera configuration files;
2. Peripheral Initialization: Keypad inputs, LED indicators, and TF card were initialized. The keypad interrupt is configured as the trigger signal for initiating image acquisition, whereas the LED indicators provide real-time status feedback (e.g., acquisition start/end).

Data Acquisition and Processing Workflow

Upon triggering, the LED indicator was illuminated to signify active acquisition. A single-frame buffering scheme was adopted to optimize memory utilization, given the system’s requirement for single-frame writes per acquisition cycle. The acquired image data were stored in DDR4 memory via a Video Direct Memory Access (VDMA) write channel.

Algorithm Execution

On the PS side, the polarization parameters—degree of polarization (Dop), polarization angle (Aop), and polarization azimuth angle (AOE)—were computed using the algorithms detailed in Section 2.1. The AOE data underwent Canny edge detection to generate a binarized edge map. The Canny algorithm is a multi-stage edge detection method designed to optimize both noise suppression and edge localization. It involves the following steps:

1. Gaussian Filtering: Smooth the input image to reduce noise;
2. Gradient Calculation: Compute intensity gradients using Sobel operators to highlight regions of high spatial derivatives;
3. Non-Maximum Suppression: Thin edges by retaining only local maxima in the gradient direction;
4. Double Thresholding: Classify pixels as strong edges, weak edges, or non-edges based on adaptive thresholds;
5. Edge Tracking: Connect weak edges to strong edges if they are contiguous, ensuring continuity.

This was followed by Hough line detection to determine the angular deviation between the solar meridian and carrier axis. The Hough transform is a feature extraction technique that converts image space lines into parameter space (polar coordinates (*ρ*, *θ*)) through a voting procedure. For each edge pixel (*x*, *y*), all possible lines passing through it are represented as

(16) ρ=xcos⁡θ+ysin⁡θ

Accumulating votes in a parameter space matrix identifies dominant lines (the solar meridian) by peak detection. In this study, an Hough transform was applied to the binarized edge map to robustly extract the solar meridian orientation, even under partial occlusion or noise interference.

Output and System Management

The calculated heading angle is transmitted via a UART interface, whereas the extracted solar meridian image is archived on the TF card. The system autonomously evaluates whether a new acquisition cycle should be initiated. Upon completion, all the subsystems were terminated.

This study employs the Vitis HLS 2020.2 development tool to implement a hardware-accelerated design for the Canny edge detection algorithm. The Vitis HLS enables FPGA-based hardware acceleration using high-level C++ programming, significantly enhancing the development efficiency. During code development, optimization directives such as pipeline insertion are applied to effectively realize data pipelining and parallel processing on the FPGA. Following simulation-based verification, the synthesized IP core was deployed into a hardware system.

In the Canny edge detection module design, AXI Master interfaces were adopted for the input/output operations. The module retrieves the memory offset addresses via the AXI-Lite interface and processes the data using the AXI-Full interface for high-throughput data transfers. Upon completion of the computations, the results are written back to memory. This architecture ensures both computational efficiency and standardized intermodule communication.

## 3. Experiment Results

### 3.1. Experimental Procedures and Results

**Figure 8:**
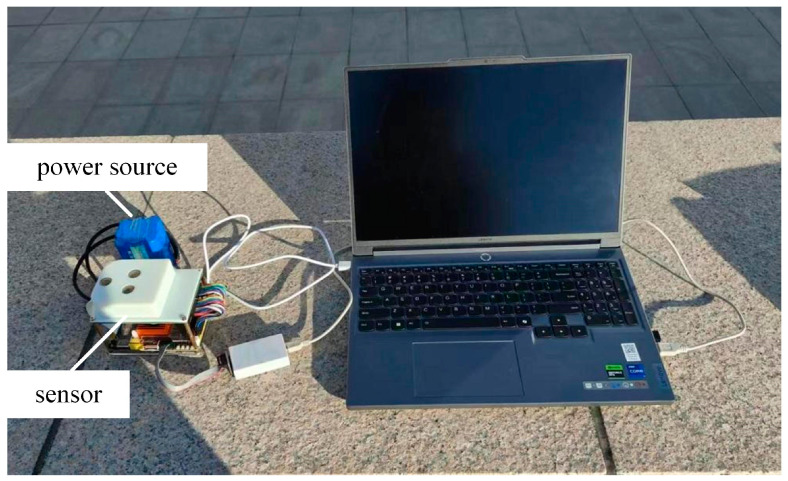
Outdoor experimental device.

**Figure 9:**
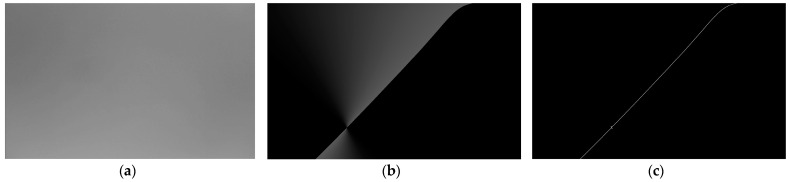
(**a**) The grayscale image collected by the sensor. (**b**) The image of polarization azimuth angle obtained through calculation. (**c**) Canny edge detection image.

The configured polarization-based heading-angle detection system was subjected to outdoor field tests. The experimental setup, depicted in Figure 8, was deployed at the western plaza of Building B in the Science Complex of the North University of China, Taiyuan, Shanxi Province (geographic coordinates: 112°26′36″ E, 38°1′1″ N). Experiments were conducted on the afternoon of 30 December 2024, under clear weather conditions. Real-time sky-polarization images were captured and processed across 44 independent trials. Figure 9a presents a representative grayscale image of the acquired sky polarization pattern, whereas Figure 9b,c illustrates the computed AOE distribution and Canny edge detection results, respectively.

**Figure 10:**
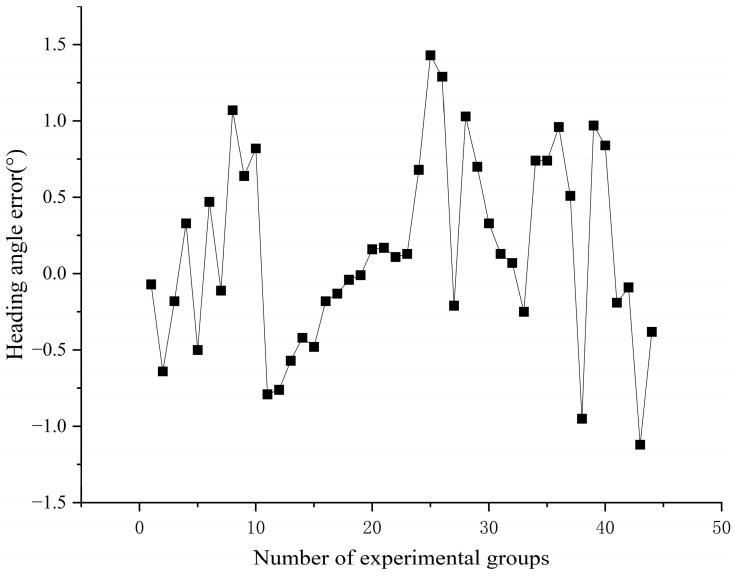
Heading angle error of the sensor. See Supplementary Materials for underlying values. (The data are shown in Table S1).

The solar meridian was extracted through a Hough transform analysis, with the heading angle determined as the angular deviation between the solar meridian and the sensor’s body axis. Figure 10 shows the quantification of the heading angle measurement errors under outdoor conditions, demonstrating the operational accuracy of the system.

### 3.2. Sources of Heading Angle Error and Comparative Analysis

The heading angle error primarily originates from the following factors:

1. Sensor Noise: Random noise introduced during polarization image acquisition by the CMOS sensor, which is attributable to hardware limitations and ambient lighting variations, degrades the polarization intensity measurement accuracy;
2. Atmospheric Interference: Fluctuations in sky polarization patterns caused by atmospheric conditions (e.g., cloud cover and aerosols) and ground-reflected light perturb polarization azimuth angle resolution;
3. Mechanical Misalignment: Angular installation deviations in the trinocular polarization camera reduce the field-of-view (FOV) overlap, compromising the multi-perspective data fusion precision.

Statistical analysis of experimental data reveals the following error characteristics:

1. Mean heading angle error: 0.50°;
2. Maximum error: 1.43°;
3. Elevated errors at low solar elevation angles (e.g., dawn/dusk) are likely associated with variations in atmospheric scattering intensity.

Performance Benchmarking

Under clear weather conditions, 50 datasets were collected to conduct comparative testing between the proposed system and PC-based processing systems. The proposed system operated at a 100 MHz clock frequency, with key performance metrics summarized in Table 1.

### 3.3. Power Consumption Measurement Methodology

To ensure the validity of the power consumption comparison between the proposed system and the PC platform, the following measurement methods were employed.

For the proposed system, power consumption of the ZYNQ-based platform was measured using a digital multimeter. The multimeter was connected in series with the 12 V DC input power supply to record current values, while operating in parallel mode to monitor voltage levels at the power terminals. Testing was conducted under full-load operation conditions. Total power consumption encompasses contributions from the ZYNQ MPSoC core, tri-channel CMOS image sensors, and auxiliary peripheral circuits including clock generators and I/O drivers. To mitigate transient fluctuations, instantaneous power values (calculated as P=V×I) were sampled at 2-s intervals over a 100-s duration, with the final results representing the average of all acquired measurements.

For the PC platform, power measurements were conducted using HWINFO (8.2.5440.0) software through its sensor monitoring interface. The procedure consisted of three phases:

1. Baseline power measurement: The system baseline was established by closing all background applications and maintaining PC idleness (no active programs). The average CPU Package Power consumption during idle state was recorded over a 5–10 min duration, denoted as P_idle;
2. Operational power measurement: MATLAB (R2023b) was launched with the target program executed. Real-time fluctuations in CPU Package Power were monitored, with the average consumption throughout the complete execution cycle recorded as P_total;
3. Program-specific power increment: The actual power attribution to the program was calculated as P_program = P_total − P_idle.

### 3.4. Key Results

- Latency: The system achieves an average heading angle output time interval of 9.43 ms (milliseconds), representing a 290.6-fold improvement over the PC system’s 2743.21 ms (milliseconds), thereby meeting real-time navigation requirements for mobile devices;
- Power Efficiency: Total power consumption was 3.6 W for the ZYNQ SoC versus 25.95 W for the PC platform, demonstrating a 7.2 times lower power consumption.

These results conclusively validate the superior performance of the system in terms of both processing speed and energy efficiency compared to conventional PC-based architectures.

As evidenced in Table 2, this study specifically addresses the demands of real-time navigation in dynamic scenarios. The heterogeneous computing framework resolves performance bottlenecks of conventional polarization sensors in mobile devices, though further optimization in accuracy remains required. Literature [13] focuses on low-cost bionic sensor design and fundamental validation, aiming to provide an economically viable polarization signal acquisition scheme for laboratory or low-speed scenarios, yet fails to address accuracy and environmental robustness. Literature [16] emphasizes integrated polarization sensors mimicking artificial compound eyes, but its complex system architecture underperforms in real-time capabilities compared to our solution. Literature [23] relies on host computer processing and high-precision sensor systems with elevated costs, prioritizing complex model computations and making them suitable for scenarios demanding extreme precision.

## 4. Conclusions

This paper presents a tri-channel integrated polarization-based heading-angle detection system centered on a ZYNQ core processor. The system employed a tri-channel polarization camera to synchronously capture sky polarization images, leveraging the parallel processing capabilities of the FPGA for hardware-accelerated image acquisition, preprocessing, and edge detection. This approach significantly reduces the system latency. Collaborative operation between the Programmable Logic (PL) and Processing System (PS) domains enhances overall computational efficiency. The experimental results demonstrate an average heading angle output time interval of 9.43 ms (milliseconds), validating the capability of the system to meet real-time processing requirements.

Furthermore, the compact design of the system ensures a minimal footprint and weight, facilitating seamless installation and deployment on mobile platforms. While maintaining robust navigation performance, this study successfully achieved sensor miniaturization and system integration. Future research will focus on optimizing the sensor performance under dynamic atmospheric conditions and expanding its applicability in complex environments, thereby advancing the practical implementation of polarization navigation technologies.

## Figures and Tables

**Table 1:** Comparative analysis of the average/maximum response time and power consumption between PC-based and Proposed Systems (the data are shown in Tables S2–S4).

Processing System	Average Response Time (ms)	Maximum Response Time (ms)	Power Consumption (W)
PC Platform	2743.21	2841.1	25.95
The Proposed System	9.43	13.89	3.6

**Table 2:** Comparison with other navigation methods.

Navigation Methods	Error	Processing System	Main Objective
The Proposed System	±0.5°	ZYNQ MPSoC	A navigation solution system with high real-time performance and low power consumption
Literature [13]	±1.53°	ARM processor	A low-cost and compact bionic polarizing sensor
Literature [16]	±0.23°	host computer	Real-time full-polarization imaging detector
Literature [23]	root mean square error 0.53°	host computer	High-precision absolute heading calculation

## Data Availability

The original contributions presented in this study are included in the article/Supplementary Material. Further inquiries can be directed to the corresponding author.

## Supplementary Materials

The following supporting information can be downloaded at https://www.mdpi.com/article/10.3390/s25092744/s1: Figure S1: Power Consumption Diagram of the Chip; Table S1: Heading Angle Error Data; Table S2: Execution Time Comparison between PC and Zynq Platforms; Table S3: PC-endurance power level; Table S4: The power consumption after the software runs.
