# Supplementary figures and images for: Real-Time Bio-Inspired Polarization Heading Resolution System Based on ZYNQ Heterogeneous Computing
Source: Sensors (Basel). 2025 Apr 26;25(9):2744. doi: 10.3390/s25092744 (PMC12074092; doi:10.3390/s25092744)

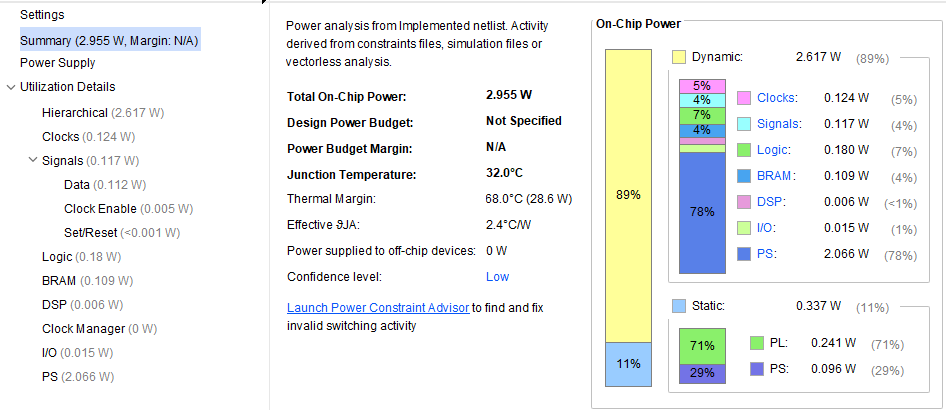

Supplement: Supplementary file 1 [file sensors-25-02744-s001.zip › Original data/Figure S1 Power Consumption Diagram of the Chip.JPEG]
